# Evaluation of Preoperative Inflammation-Based Prognostic Scores in Patients With Intrahepatic Cholangiocarcinoma: A Multicenter Cohort Study

**DOI:** 10.3389/fonc.2021.672607

**Published:** 2021-06-17

**Authors:** Chaobin He, Chongyu Zhao, Jiawei Lu, Xin Huang, Cheng Chen, Xiaojun Lin

**Affiliations:** ^1^ Department of Pancreatobiliary Surgery, State Key Laboratory of Oncology in South China, Collaborative Innovation Center for Cancer Medicine, Sun Yat-sen University Cancer Center, Guangzhou, China; ^2^ Department of Oncology, The Second Hospital of Dalian Medical University, Dalian, China; ^3^ Department of Cardiology, The First Affiliated Hospital of Dalian Medical University, Dalian, China

**Keywords:** intrahepatic cholangiocarcinoma, Modified Glasgow Prognostic Scores, overall survival, progression-free survival, prognosis

## Abstract

**Background:**

Accumulating evidence has indicated the vital role of inflammation-based score (IBS) in predicting the prognostic outcome of cancer patients. Otherwise, their value in intrahepatic cholangiocarcinoma (iCCA) remains indistinct. The present study aimed to evaluate whether IBSs were related to survival outcomes in iCCA patients.

**Method:**

Clinical characteristics were retrospectively collected in 399 patients diagnosed with iCCA from cohorts of Sun Yat-sen University Cancer Center (SYSUCC) and the First Hospital of Dalian Medical University (FHDMU). The survival curves were constructed with the Kaplan-Meier method and compared with the log-rank test. Univariate and multivariate analyses were conducted to determine the prognostic factors of overall survival (OS) and progression-free survival (PFS). The concordance index and the area under the time-dependent receiver operating characteristic (ROC) curves (AUROCs) were used to compare the predictive value of inflammation-based scores in terms of survival outcomes.

**Results:**

The significant survival differences in OS and DFS were observed when patients were stratified by the modified Glasgow Prognostic Score (mGPS) (p<0.001). Multivariate analysis demonstrated that higher mGPS score was independently associated with poor OS and DFS (p<0.001). The predictive accuracy of the mGPS was superior to other IBSs (all p<0.001) in survival prediction in iCCA patients. The findings were further supported by the external validation cohort.

**Conclusion:**

The mGPS is a sensitive, efficient, simple and widely applicable preoperative prognostic factor for iCCA patients. Thus, more effective therapy and frequent surveillance should be conducted after surgical resection in iCCA patients with higher mGPS scores.

## Background

Intrahepatic cholangiocarcinoma (iCCA) is the second most common malignant tumor ranking after hepatocellular carcinoma (1). Although iCCA patients in different stages can be treated with various modalities, including surgery resection, chemotherapy, and radiation therapy, the overall incidence and mortality have shown a worldwide increase in the past decades (1, 2). Even though surgical resection provided the best chances to obtain prolonged survival, the median progression-free survival (PFS) time was reported to be merely 12 to 36 months in patients with resectable iCCA (3). To optimize risk-benefit assessments and stratify the patients for more individualized treatment, there is an urgent demand to seek an objective, sensitive and reliable prognostic marker for patients with iCCA. Currently, common prognostic markers, such as tumor margins, tumor differentiation, and lymph node metastases, are determined only after surgical resection (2). Therefore, there is continuing momentum in finding a practical pre-operative prognostic marker that could facilitate accurate patient stratification before surgery and improve therapeutic outcomes.

Inflammation, as a new hall marker of cancer (4), plays a vital role in the progression of tumors (5). Tumors produce inflammatory chemokines and cytokines and are locally infiltrated by leucocytes (6). Moreover, the activation of the ongoing systemic chronic inflammatory response will further lead to cachexia (6). According to these pieces of evidence, many inflammation-based scores (IBSs) were proved to be prognostic in various tumors, including Glasgow Prognostic Score (GPS) and modified Glasgow Prognostic Score (mGPS) (7), Prognostic Index (PI) (8), Prognostic Nutritional Index (PNI) (9), systemic immune-inflammation index (SII) (10), neutrophil to lymphocyte ratio (NLR) (11), platelet to lymphocyte ratio (PLR) (12), and lymphocyte to monocyte ratio (LMR) (13). Nonetheless, the research about reliable and valid inflammation-based scores in patients with iCCA after resection remains supplemented. Besides, most previous studies were conducted in a single center with a small number of patients and were mostly concentrated on a certain single IBS (14–17). Thus, for evaluating the validity of the IBSs in iCCA patients, a multicenter study with a large volume of patients would be necessary and imperative. According to these findings, our study aimed to find the best combination of inflammatory factors that could predict survival outcomes for iCCA patients after surgical resection.

## Methods

### Study Design and Patient Materials

**Figure 1:**
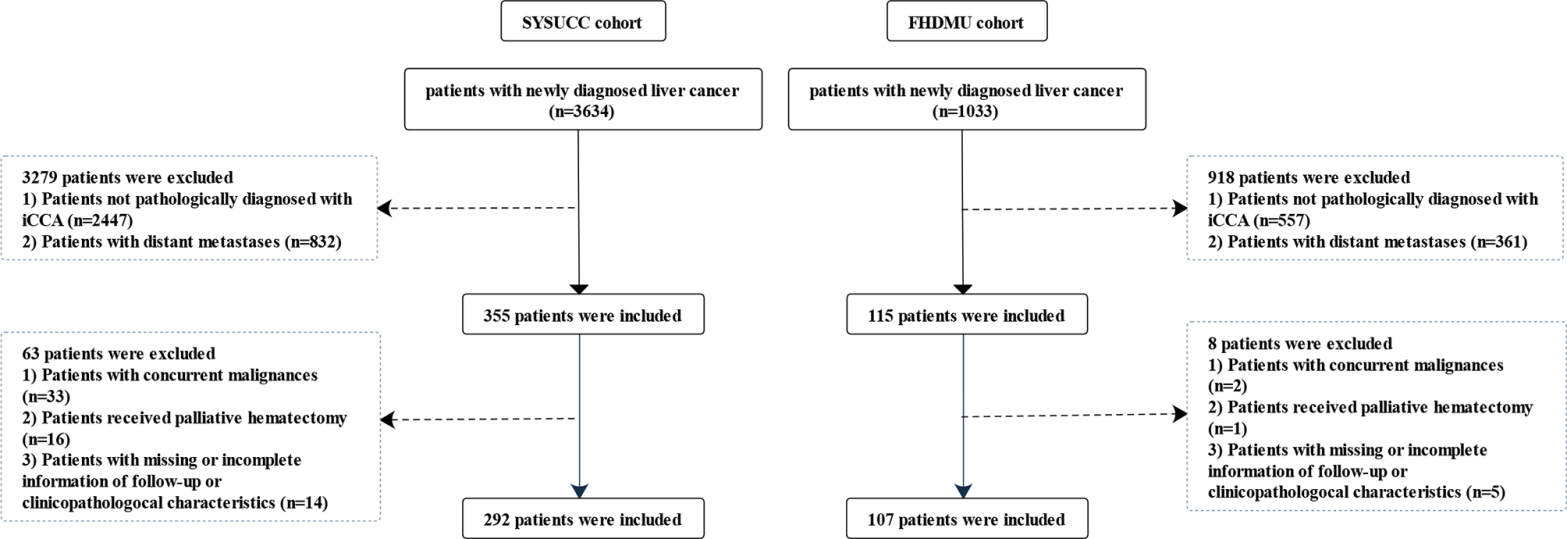
Flow chart of the patient enrolling process.

A total of 399 patients pathologically diagnosed with iCCA from two cohorts were finally enrolled in the present study [292 patients from Sun Yat-sen University Cancer Center (SYSUCC) between January 2000 and December 2018 as the primary cohort and another 107 patients from the first affiliated hospital of Dalian Medical University (FHDMU) between May 2013 and December 2019 as the validation cohort]. The enrolling flowchart of patients was presented in **Figure 1**. Clinical characteristics were retrospectively aggregated from the electronic medical record and were exhibited in **Table 1**. This study obtained the written informed consent from all the patients and was approved by the ethics committees of two participating centers.

**Table 1:** Clinical, radiological, and pathological characteristics of the SYSUCC cohort and FHDMU cohort.

Variables	Primary cohort (n = 292)	Validation cohort (n = 107)	Variables	Primary cohort (n = 292)	Validation cohort (n = 107)
Gender			PI		
Male	181 (62.0%)	62 (57.9%)	0	220 (75.3%)	32 (29.9%)
Female	111 (38.0%)	45 (42.1%)	1	61 (20.9%)	63 (58.9%)
Age (years)			2	11 (3.8%)	12 (11.2%)
≤60	189 (64.7%)	33 (30.8%)	Tumor capsular		
>60	103 (35.3%)	74 (69.2%)	Absence	45 (15.4%)	–
WBC count (×10^9^/L)			Uncompleted	37 (12.7%)	–
≤10	259 (88.7%)	92 (86.0%)	Completed	210 (71.9%)	–
>10	33 (11.3%)	15 (14.0%)	Satellite sites		
HGB (g/L)			Absence	201 (68.8%)	106 (99.1%)
≤175	27 (9.20%)	30 (28.0%)	Presence	91 (31.2%)	1 (0.90%)
>175	265 (90.8%)	77 (72.0%)	Thrombus		
PLT (×10^9^/L)			Absence	269 (92.1%)	–
≤350	10 (3.40%)	5 (4.70%)	Presence	23 (7.90%)	–
>350	282 (96.6%)	102 (95.3%)	Tumor differentiation		
ALT (U/L)			Low	6 (2.10%)	3 (2.80%)
≤50	236 (80.8%)	55 (51.4%)	Medium	105 (35.9%)	81 (75.7%)
>50	56 (19.2%)	52 (48.6%)	High	181 (62.0%)	23 (21.5%)
AST (U/L)			Microvascular invasion		
≤40	254 (87.0%)	56 (52.3%)	Absence	237 (81.2%)	86 (89.7%)
>40	38 (13.0%)	51 (47.7%)	Presence	55 (18.8%)	11 (10.3%)
GGT (U/L)			Lymph-vessel invasion		
≤60	108 (37.0%)	16 (15.0%)	Absence	273 (93.5%)	–
>60	184 (63.0%)	91 (85.0%)	Presence	19 (6.5%)	–
ALP (U/L)			Macro vascular invasion		
≤125	182 (62.3%)	25 (23.4%)	Absence	274 (93.8%)	95 (88.8%)
>125	110 (37.7%)	82 (76.6%)	Presence	18 (6.20%)	12 (11.2%)
ALB (g/L)			Back membrane invasion		
>40	5 (1.70%)	38 (35.5%)	Absence	114 (39.0%)	90 (84.1%)
≤40	287 (98.3%)	69 (64.5%)	Presence	178 (61.0%)	12 (15.9%)
TBIL (μmol/L)			Imaging tumor size		
≤20.5	265 (90.8%)	54 (50.5%)	≤5 cm	131 (44.9%)	56 (52.3%)
>20.5	27 (9.20%)	53 (49.5%)	≤5 cm	161 (55.1%)	51 (47.7%)
IBIL (μmol/L)			Imaging vascular invasion		
≤15	275 (94.2%)	65 (60.7%)	Absence	271 (92.8%)	97 (90.7%)
>15	17 (5.80%)	42 (39.3%)	Presence	21 (7.20%)	10 (9.30%)
CRP (mg/L)			Imaging LN metastasis		
≤3	172 (58.9%)	35 (32.7%)	Absence	207 (70.9%)	54 (50.5%)
>3	120 (41.1%)	72 (67.3%)	Presence	85 (29.1%)	53 (49.5%)
HBsAg			Imaging LN size		
Absence	162 (55.5%)	105 (98.1%)	Absence	207 (70.9%)	–
Presence	130 (44.5%)	2 (1.9%)	≤1 cm	28 (9.60%)	–
CA19-9 (U/ml)			>1 cm	57 (19.5%)	–
≤35	141 (48.3%)	25 (23.4%)	Tumor size		
>35	151 (51.7%)	82 (76.6%)	≤5 cm	115 (39.4%)	52 (48.6%)
CEA (ng/ml)			≤5 cm	177 (60.6%)	55 (51.4%)
≤5	211 (72.3%)	60 (56.1%)	LN metastasis		
>5	81 (27.7%)	47 (43.9%)	Absence	250 (85.6%)	95 (88.8%)
LCR			Presence	42 (14.4%)	12 (11.2%)
0	21 (7.20%)	–	Nerve tract invasion		
1	271 (92.8%)	–	Absence	96 (89.7%)	96 (89.7%)
mGPS			Presence	11 (10.3%)	11 (10.3%)
0	216 (74.0%)	37 (34.6%)	Peri-origin invasion		
1	67 (22.9%)	43 (40.2%)	Absence	269 (92.1%)	103 (96.3%)
2	9 (3.10%)	27 (25.2%)	Presence	23 (7.90%)	4 (3.70%)
NLR			T stage 8th		
<2.62	194 (66.4%)	36 (33.6%)	1	34 (24.3%)	84 (78.5%)
≥2.62	98 (33.6%)	71 (66.4%)	2	44 (15.1%)	5 (4.7%)
LMR			3	153 (52.4%)	14 (13.1%)
<4.06	125 (42.8%)	–	4	24 (8.20%)	4 (3.7%)
≥4.06	167 (57.2%)	–	N stage 8^th^		
PLR			Absence	250 (85.6%)	95 (88.8%)
<104.85	172 (58.9%)	24 (22.4%)	Presence	42 (14.4%)	12 (11.2%)
≥104.85	120 (41.1%)	83 (77.6%)	TNM 8^th^		
SII			I	70 (24.0%)	81 (75.7%)
0	68 (23.3%)	30 (28.0%)	II	37 (12.7%)	2 (1.90%)
1	224 (76.7%)	77 (72.0%)	III	185 (63.4%)	24 (22.4%)
PNI			After operation therapy		
0	277 (94.9%)	48 (44.9%)	Absence	161 (55.1%)	72 (67.3%)
1	15 (5.1%)	59 (55.1%)	Presence	131 (44.9%)	35 (32.7%)

WBC, white blood cell; HGB, hemoglobin; PLT, platelets; ALT, alanine aminotransferase; AST, glutamic-oxalacetic transaminase; GGT, gamma-glutamyl transpeptidase; ALP, alkaline phosphatase; ALB, Albumin; TBIL, total serum bilirubin; IBIL, indirect serum bilirubin; CRP, C-reaction protein; CA19-9, carbohydrate antigen 19-9; CEA, carcinoembryonic antigen; LCR, lymphocyte-C-reactive protein ratio; mGPS, modified Glasgow prognostic score; NLR, neutrophil-lymphocyte ratio; LMR, lymphocyte to monocyte ratio; PLR, platelet to lymphocyte ratio; SII, systemic immune-inflammation index; PNI, prognostic nutritional index; PI, prognostic Index; LN, lymph node.

### Survival Outcomes and Follow-Up

The study’s outcome variables, overall survival (OS) and PFS, were calculated from the date of surgery to the date of death and tumor progression, respectively, or the last follow-up. The first post-operative follow-up was conducted at 30 days after surgical resection, then every three months for the first year, and every six months until death or dropout. Follow-up data of two cohorts were retrieved on November 30, 2020.

### Standard Management of iCCA Patients

The indications to resection and contraindications were the same in two centers of this study. The following indications to resection were followed: 1) Clinically diagnosed with iCCA according to the laboratory measurements and the imaging examinations. 2) The tumor was resectable. 3) No distant lymph-node metastasis or distant organ metastasis were observed. The contraindications included inoperable cardiopulmonary dysfunction, large volume of ascites and cachexy. Preoperative blood samples were routinely collected 1 week before surgery or at the preoperative outpatient visit. Routine laboratory measurements of differential leukocyte count and classification, including C-reactive protein (CRP), hemoglobin (HGB), platelet (PLT), tumor biomarkers (alpha-fetoprotein [AFP], carbohydrate antigen 19-9 [CA19-9], carcinoma embryonic antigen [CEA]) and blood biochemistry (serum albumin [ALB], alanine transaminase [ALT], glutamic-oxalacetic transaminase [AST], alkaline phosphatase [ALP], gamma-glutamyl transpeptidase [GGT], indirect bilirubin [IBIL], and total bilirubin [TBIL]) were carried out. The preoperative imaging evaluations for iCCA included abdomen computed tomography (CT), chest CT, pelvis CT and magnetic resonance imaging. Those patients with jaundice or dilated bile ducts routinely underwent biliary drainage. Once the regional LN metastasis was implicated in the preoperative imaging evaluations or suspected during surgery, all resectable regional LNs were dissected. The postoperative pathological stage of iCCA was classified according to the eighth AJCC TNM staging system. Moreover, the adjuvant chemotherapy was routinely implemented in the patients with more advanced or aggressive tumors, particularly those with LN metastasis.

### Inflammation-Based Scores

According to our previous study (18, 19) related to the survival predicting performance of IBSs, NLR, PLR, LCR, LMR, PI, GPS, mGPS, PNI, and SII were included and calculated in this multicohort study to identify the IBS with highest accuracy to predict poor OS and PFS in iCCA patients. The details of IBSs were described in **Table 2**.

**Table 2:** Inflammation-based prognostic scoring systems.

Scoring systems	Score
The modified Glasgow Prognostic Score (mGPS)	
CRP (≤10 mg/L) and albumin (≥35 g/L)	0
CRP (≤10 mg/L) and albumin (<35 g/L)	0
CRP (>10 mg/L) and albumin (≥35 g/L)	1
CRP (>10 mg/L) and albumin (<35 g/L)	2
Lymphocyte-C-reactive Protein ratio (LCR)	
10^4^ × lymphocyte count (10^9^/L): CRP (mg/L) >6000	0
10^4^ × lymphocyte count (10^9^/L): CRP (mg/L) ≤6000	1
Neutrophil to lymphocyte ratio (NLR)	
Neutrophil count: lymphocyte count < 5:1	0
Neutrophil count: lymphocyte count ≥ 5:1	1
Lymphocyte to monocyte ratio (LMR)	
Lymphocyte count (×10^9^/L): monocyte count (×10^9^/L) <3	0
Lymphocyte count (×10^9^/L): monocyte count (×10^9^/L) ≥3	1
Platelet to lymphocyte ratio (PLR)	
Platelet count: lymphocyte count < 150:1	0
Platelet count: lymphocyte count ≥ 150:1	1
Platelet count: lymphocyte count > 300:1	2
Systemic immune-inflammation index (SII)	
Platelet count (×10^9^/L) × neutrophil count (×10^9^/L)/lymphocyte count (×10^9^/L) < 305	0
Platelet count (×10^9^/L) × neutrophil count (×10^9^/L)/lymphocyte count (×10^9^/L) ≥ 305	1
Prognostic Nutritional Index (PNI)	
Albumin (g/L) +5 × total lymphocyte count (10^9^/L) ≥45	0
Albumin (g/L) +5 × total lymphocyte count (10^9^/L) <45	1
Prognostic index (PI)	
CRP (≤10 mg/L) and white blood cell count (≤11 × 10^9^/L)	0
CRP (≤10 mg/L) and white blood cell count (>11 × 10^9^/L)	1
CRP (>10 mg/L) and white blood cell count (≤11 × 10^9^/L)	1
CRP (>10 mg/L) and white blood cell count (>11 × 10^9^/L)	2

Abbreviations as in **Table 1**.

### Statistical Analysis

Continuous variables were reported with median and interquartile range. Categorical variables were reported with whole numbers and proportions. Proportions were compared using the chi-square test or the Fisher Exact test. Distributions of continuous variables were compared using the Mann-Whitney U test. Maximally selected rank statistic from the R package was employed to identify the optimal cutoff points of NLR, PLR and LMR. Survival curves were generated using the Kaplan-Meier method and compared with the log-rank test. The Cox regression model was used to perform the multivariate analysis of the predictive factors of OS and PFS. Time-dependent receiver operating characteristic curves (ROC) were analyzed to compare the prognostic ability of these eight inflammation-based scores. The concordance index (C-index) and the area under the ROC curves (AUROCs) were performed using R software version 3.5.0 (The R Foundation for Statistical Computing, Vienna, Austria. http://www.rproject.org). All statistical inferences were based on two-sided p values, with values <0.05 taken to indicate statistical significance.

## Results

### Patient Characteristics

A total of 399 patients pathological diagnosed with iCCA from two different patient cohorts were enrolled in this study. In the primary cohort, 181 male (62.0%) and 62 female (57.9%) iCCA patients with a median age of 56 years (range, 20–77 years) were enrolled. There were 70 (24.0%) patients diagnosed as TNM stage I, 37 (12.7%) patients as stage II, and 185 (63.4%) as stage III, respectively. Moreover, a majority of patients were assigned into LCR 0 (271, 92.8%), NLR< 2.62 (194, 66.4%), LMR≥ 4.06 (167, 57.2%), PLR< 104.85 (172, 58.9%), SII 1 (224, 76.7%), PNI 0 (277, 94.9%), and PI 0 (220, 75.3%), respectively. Specially, 216 (74.0%) patients had an mGPS of 0, 67 (22.9%) patients had an mGPS of 1, and 9 patients (3.1%) had an mGPS of 2. The validation cohort consisted of 62 males (57.9%) and 45 females (42.1%) with a median age of 64 years (range, 32–88 years). Slightly different from the primary cohort, a majority of patients were assigned into NLR≥2.62 (71, 66.4%), PLR≥ 104.85 (83, 77.6%), SII 1 (77, 72.0%), PNI 1 (59, 55.1%), and PI 1 (63, 58.9%) in the validation cohort, respectively. No significant differences were observed in baseline characteristics between the included patients. Further hematologic, imaging and pathological characteristics are presented in **Table 1**.

### Survival Outcomes According to IBSs

**Figure 2:**
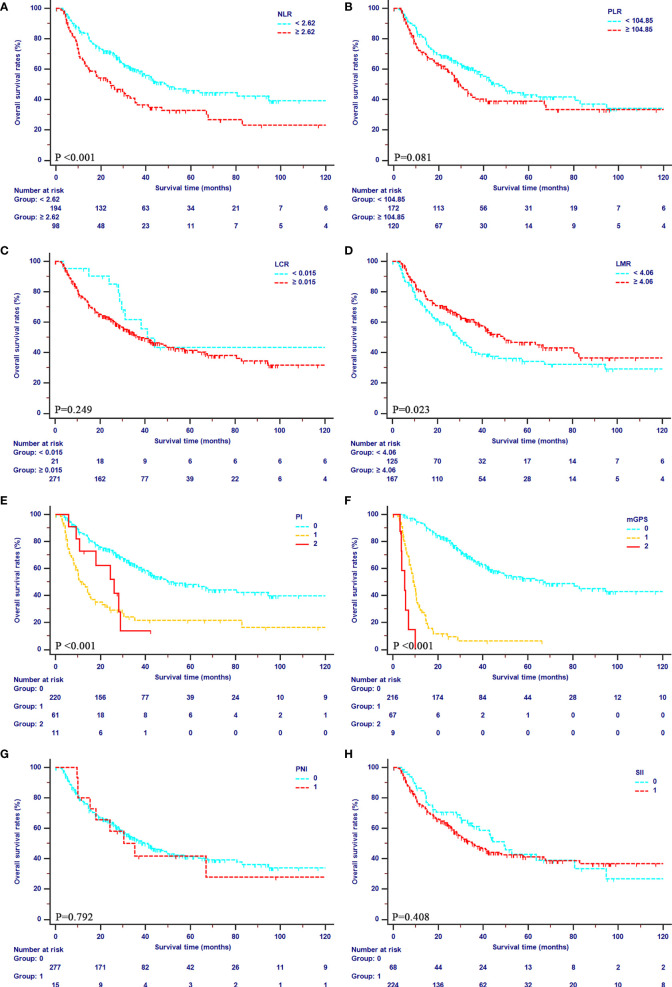
Kaplan-Meier curves for OS in patients with iCCA in the SYSUCC cohort stratified by the inflammation-based score systems. **(A)**, NLR; **(B)**, PLR; **(C)**, LCR; **(D)**, LMR; **(E)**, PI; **(F)**, mGPS; **(G)**, PNI; **(H)**, SII.

**Figure 3:**
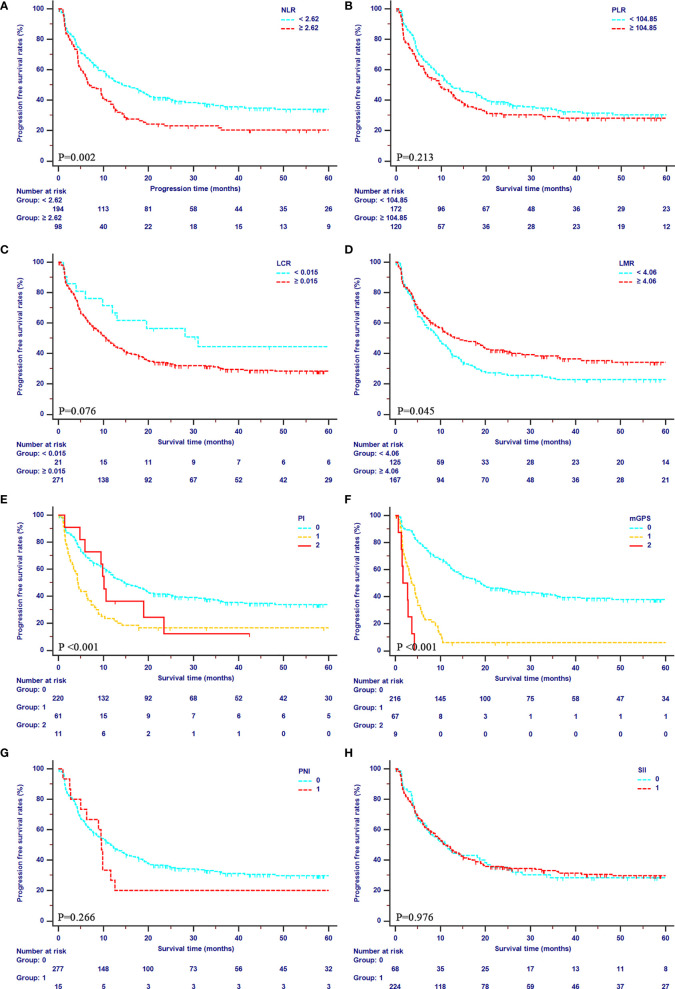
Kaplan-Meier curves for PFS in patients with iCCA in the SYSUCC cohort stratified by the inflammation-based score systems. **(A)**, NLR; **(B)**, PLR; **(C)**, LCR; **(D)**, LMR; **(E)**, PI; **(F)**, mGPS; **(G)**, PNI; **(H)**, SII.

The median OS of patients were 39.47 months (95% CI, 31.03–49.87 months) in the primary cohort, and 16.23 months (95% CI, 12.23–24.10 months) in the validation cohort, respectively. The median PFS was 11.23 months (95% CI, 8.87–14.13 months) in the primary cohort, and 12.87 months (95% CI, 10.10–16.97 months) in the validation cohort, respectively. In the primary cohort, mGPS showed an outstanding prediction of both OS (1-year OS rates: 94.4%, 29.2% and 0%; 2-year OS rates: 81.8%, 11.7%, and 0%; 3-year OS rates: 65.8%, 6.23%, 0%) and PFS (1-year PFS rates: 62.9%, 6.1%, and 0%; 2-year PFS rates: 45.8%, 6.1%, and 0%; 3-year PFS rates: 39.4%, 6.1%, 0%). Additionally, poor OS was obtained in patients with higher values of PI and NLR (all P<0.001) and lower values of LMR (P=0.023). Meanwhile, patients with higher values of PI (P<0.001), NLR (P=0.002), and lower values of LMR (P=0.045) showed poor PFS as well. All the details of OS curve and PFS curves in the primary cohort were shown in **Figures 2**, **3**, respectively.

### Prognostic Factors for Survival Outcomes

For our primary cohort, the univariate analysis identified 22 hematological, pathological, and radiological elements and IBSs as prognostic factors for OS and PFS (**Table 2**). Additionally, the Cox-regression analysis was conducted to distinguish the independent risk factors of OS and PFS. In the multivariate analysis, only CA19-9 (HR, 1.568; 95% CI, 1.071–2.296; P = 0.021), CEA (HR, 1.677; 95% CI, 1.112–2.528; P = 0.014), mGPS (HR, 37.929; 95% CI, 12.609–113.367; P < 0.001), PI (HR, 0.187; 95% CI, 0.059–0.593; P = 0.004), imaging 9^th^ LN metastasis (HR, 3.179; 95% CI, 1.092–9.256; P = 0.034), and after operation therapy (HR, 1.941; 95% CI, 1.345–2.778; P < 0.001) displayed statistical difference of OS, and the factors independently associated with PFS were: CA19-9 (HR, 1.586; 95% CI, 1.140–2.208; P = 0.006), mGPS (P < 0.001), PI (P < 0.001), imaging LN metastasis (HR, 1.462; 95% CI, 1.168–1.829; P < 0.001), and after operation therapy (HR, 3.571; 95% CI, 1.878–5.150; P < 0.001) (**Table 3**).

**Table 3:** Univariate and multivariate analyses of prognostic factors of OS and PFS in the SYSUCC cohort.

Variables	OS				PFS			
	Univariate		Multivariate		univariate		multivariate	
	HR (95% CI)	P	HR (95% CI)	P	HR (95% CI)	P	HR (95% CI)	P
Gender (Male: Female)	0.853 (0.610–1.192)	0.351			0.778 (0.581–1.043)	0.093		
Age, years (≤60:>60)	1.137 (0.818–1.579)	0.445			1.007 (0.756–1.340)	0.964		
WBC, ×10^9^/L (≤10: >10)	2.395 (1.551–3.697)	<0.001	1.850 (0.962–3.557)	0.065	1.541 (1.019–2.329)	0.040	1.864 (0.972–3.574)	0.061
HGB, g/L (≤:175 >175)	1.455 (0.787–2.691)	0.232			1.258 (0.765–2.069)	0.365		
PLT, ×10^9^/L (≤350: >350)	0.844 (0.373–1.911)	0.684			1.159 (0.545–2.465)	0.701		
ALT, U/L (≤50: >50)	1.443 (0.984–2.117)	0.061			1.225 (0.874–1.717)	0.239		
AST, U/L (≤40: >40)	1.266 (0.805–1.991)	0.307			1.315 (0.896–1.931)	0.162		
GGT, U/L (≤60: >60)	2.003 (1.393–2.879)	<0.001	0.980 (0.618–1.554)	0.931	1.518 (1.126–2.045)	0.006	0.780 (0.530–1.147)	0.206
ALP, U/L (≤125: >125)	2.583 (1.868–3.573)	<0.001	1.194 (0.755–1.890)	0.448	1.864 (1.410–2.463)	<0.001	1.166 (0.792–1.717)	0.435
ALB, g/L (≥35:<35)	1.086 (0.877–1.344)	0.449			0.986 (0.794–1.225)	0.899		
TBIL, μmol/L (≤20.5: >20.5)	1.361 (0.821–2.256)	0.232			1.222 (0.781–1.911)	0.380		
IBIL, μmol/L (≤15: >15)	1.139 (0.599–2.166)	0.691			1.492 (0.873–2.549)	0.143		
CRP, mg/L (≤3: >3)	2.072 (1.499–2.863)	<0.001	0.879 (0.550–1.405)	0.590	2.015 (1.524–2.664)	<0.001	1.229 (0.858–1.760)	0.260
HBsAg (no: yes)	1.012 (0.731–1.402)	0.940			1.180 (0.894–1.557)	0.243		
CA19-9, U/ml (≤35: >35)	1.951 (1.402–2.714)	<0.001	1.568 (1.071–2.296)	0.021	1.939 (1.459–2.575)	<0.001	1.586 (1.140–2.208)	0.006
CEA, ng/ml (≤5: >5)	2.713 (1.940–3.792)	<0.001	1.677 (1.112–2.528)	0.014	1.756 (1.301–2.370)	<0.001	0.898 (0.621–1.299)	0.568
LCR (0: 1)	1.458 (0.766–2.776)	0.249			1.723 (0.937–3.168)	0.076		
mGPS								
0	Ref		Ref		Ref		Ref	
1	9.902 (6.758–14.510)	<0.001	12.609 (7.142–21.251)	<0.001	4.548 (3.278–6.308)	<0.001	4.128 (2.634–6.489)	<0.001
2	41.983 (17.84–98.802)	<0.001	37.929 (12.609–113.367)	<0.001	11.709 (5.482–25.009)	<0.001	5.417 (2.1–13.976)	<0.001
NLR (<2.62: ≥2.62)	1.763 (1.271–2.446)	<0.001	0.890 (0.573–1.382)	0.604	1.562 (1.175–2.076)	0.002	0.959 (0.652–1.410)	0.831
LMR (<4.06: ≥4.06)	0.691 (0.501–0.953)	0.023	0.878 (0.591–1.304)	0.518	0.754 (0.571–0.995)	0.045	0.920 (0.647–1.308)	0.642
PLR (<104.85: ≥104.85)	1.332 (0.963–1.843)	0.081			1.194 (0.903–1.580)	0.213		
SII (0: 1)	1.175 (0.801–1.724)	0.408			1.005 (0.725–1.393)	0.976		
PNI (0: 1)	1.095 (0.558–2.149)	0.792			1.374 (0.782–2.414)	0.266		
PI								
0	Ref		Ref		Ref		Ref	
1	3.092 (2.157–4.433)	<0.001	0.896 (0.510–1.575)	0.703	2.146 (1.549–2.972)	<0.001	0.776 (0.478–1.259)	0.304
2	2.458 (1.189–5.083)	0.015	0.187 (0.059–0.593)	0.004	1.442 (0.734–2.833)	0.289	0.140 (0.048–0.414)	<0.001
Imaging tumor size (≤5 cm: >5 cm)	1.913 (1.368–2.676)	<0.001	1.011 (0.648–1.577)	0.961	1.758 (1.322–2.338)	<0.001	1.170 (0.785–1.744)	0.441
Imaging vascular invasion (no: yes)	1.178 (0.942–1.474)	0.151			1.239 (0.987–1.555)	0.065		
Imaging LN metastasis								
5^th^ LN metastasis	0.049 (0–237.011)	0.486			0.383 (0–49.654)	0.043	0 (0–7.198 × 10^144^)	0.946
7^th^ LN metastasis	1.031 (0.255–4.171)	0.965			1.476 (0.536–4.062)	0.451		
8^th^ LN metastasis	1.675 (0.976–2.877)	0.061			1.421 (0.881–2.290)	0.150		
9^th^ LN metastasis	3.177 (1.292–7.815)	0.012	3.179 (1.092–9.256)	0.034	2.294 (1.016–5.181)	0.046	1.112 (0.412–2.907)	0.834
12^th^ LN metastasis	2.847 (2.030–3.994)	<0.001	1.272 (0.740–2.185)	0.384	2.714 (1.930–3.817)	<0.001	1.936 (1.146–3.272)	0.014
13^th^ LN metastasis	1.752 (0.773–3.970)	0.179			1.842 (0.906–3.744)	0.092		
14^th^ LN metastasis	0.049 (0–4335.171)	0.667			3.235 (0.450–23.26)	0.243		
16^th^ LN metastasis	2.570 (0.917–8.083)	0.106			2.381 (0.758–7.475)	0.137		
Imaging LN size								
Absence	Ref		Ref		Ref		Ref	
≤1 cm	1.616 (0.987–2.645)	0.056	1.032 (0.422–1.185)	0.521	1.530 (0.964–2.428)	0.071	1.450 (0.724–2.312)	0.141
>1 cm	1.948 (1.312–2.893)	0.001	1.872 (0.671–2.881)	0.471	2.188 (1.576–3.038)	<0.001	2.528 (0.672–4.138)	0.341
Tumor capsular (no: yes)	1.003 (0.811–1.240)	0.979			1.018 (0.844–1.226)	0.854		
Satellite sites (no: yes)	1.123 (1.044–1.208)	<0.001	0.946 (0.554–1.615)	0.839	2.147 (1.612–2.860)	<0.001	1.423 (0.923–2.195)	0.110
Thrombus (no: yes)	1.802 (1.087–2.987)	0.022	1.327 (0.736–2.363)	0.336	1.516 (0.955–2.406)	0.078		
Tumor differentiation								
Well	Ref				Ref		Ref	
Moderate	2.172 (0.528–8.930)	0.282			2.810 (0.688–11.484)	0.150	3.668 (0.788–17.080)	0.098
Poor	2.779 (0.682–11.326)	0.154			3.733 (0.922–15.119)	0.065	4.440 (0.957–20.644)	0.057
Microvascular invasion (no: yes)	1.606 (1.078–2.392)	0.020			1.754 (1.249–2.462)	<0.001	1.167 (0.750–1.815)	0.493
Lymph-vessel invasion (no: yes)	1.477 (0.851–2.563)	0.166			1.239 (0.732–2.097)	0.425		
Macrovascular invasion (no: yes)	1.530 (0.828–2.829)	0.175			1.419 (0.838–2.402)	0.193		
Adjacent organ invasion included gallbladder (no: yes)	1.765 (1.111–2.804)	0.016	0.608 (0.205–1.802)	0.369	1.902 (1.280–2.826)	<0.001	1.787 (0.750–4.258)	0.190
LN metastasis (no: yes)	3.304 (2.251–4.850)	<0.001	0.872 (0.427–1.784)	0.709	3.078 (2.158–4.392)	<0.001	0.901 (0.475–1.707)	0.748
Liver capsule invasion (no: yes)	1.240 (0.889–1.730)	0.205	0.418 (0.314–1.306)	0.134	1.391 (1.042–1.858)	0.025	1.093 (0.475–2.516)	0.835
T stage 8^th^								
1	Ref		Ref		Ref		Ref	
2	2.079 (1.231–3.512)	0.006	1.519 (0.752–3.16)	0.312	1.717 (1.078–2.735)	0.023	1.546 (1.756–4.237)	0.543
3	1.781 (1.153–2.751)	0.009	1.721 (0.101–5.317)	0.724	1.764 (1.226–2.539)	0.002	1.412 (0.428–4.186)	0.142
4	2.482 (1.315–4.686)	0.005	1.892 (0.698–7.972)	0.811	2.207 (1.296–3.758)	0.004	1.394 (0.334–4.257)	0.610
Tumor size (≤5 cm: >5 cm)	2.900 (1.997–4.211)	<0.001	1.301 (0.753–2.248)	0.345	2.164 (1.605–2.916)	<0.001	1.396 (0.903–2.158)	0.134
TNM 8^th^								
I	Ref		Ref		Ref		Ref	
II	1.602 (0.898–2.857)	0.111	2.571 (0.261–25.445)	0.419	1.362 (0.819–2.267)	0.234	1.519 (0.162–14.205)	0.714
III	2.048 (1.335–3.141)	0.001	2.968 (0.327–26.925)	0.334	1.943 (1.360–2.777)	<0.001	2.540 (0.292–22.100)	0.398
After operation therapy (no: yes)	1.901 (1.372–2.635)	<0.001	1.941 (1.345–2.778)	<0.001	3.176 (2.3678–4.260)	<0.001	3.571 (1.878–5.150)	<0.001

OS, overall survival; PFS, progression-free survival; Ref, reference. Other abbreviations as in **Table 1**.

### The External Validation of Significant Prognostic Factors

According to the statistic results in the primary cohort, an external validation was conducted. The significant prognostic factors, which were defined in the primary cohort, were validated in the FHDMU cohort. The multivariate analysis based on the validation cohort indicated that only mGPS was an independent prognostic factor for both OS and PFS (**Table 4**). In addition, survival was also well separated by mGPS in the external validation cohort (OS, 1-year rates: 79.8%, 52.2%, and 45.4%; 2-year rates: 63.9%, 24.2%, and 15.2%; 3-year rates: 60.1%, 16.1%, 12.6%; P < 0.001; PFS, 1-year rates: 66.3%, 42.4%, and 45.6%; 2-year rates: 51.9%, 23.0%, and 16.7%; 3-year rates: 39.7%, 11.5%, 8.34%; P = 0.003) (**Supplementary Figure 1**).

**Table 4:** External validation of significant prognostic factors in primary cohort.

Variables	OS		DFS	
	HR (95% CI)	P	HR (95% CI)	P
CA19-9, U/ml (≤35: >35)	0.663 (0.354–1.240)	0.198	0.823 (0.484–1.402)	0.474
CEA, ng/ml (≤5: >5)	1.734 (0.983–3.059)	0.057	1.134 (0.763–1.588)	0.631
mGPS				
0	Ref		Ref	
1	6.563 (2.024–21.28)	0.002	6.763 (2.445–18.705)	< 0.001
2	10.598 (2.994–37.514)	< 0.001	9.128 (3.023–27.564)	< 0.001
Imaging LN metastasis (no: yes)	1.141 (0.679–1.917)	0.618	1.317 (0.829–2.092)	0.243
After operation therapy (no: yes)	0.734 (0.430–1.251)	0.256	0.738 (0.456–1.195)	0.217

Abbreviations as in **Table 3**.

### Comparison of the Predictive Power of IBSs on Survival Outcomes in Two Cohorts

**Figure 4:**
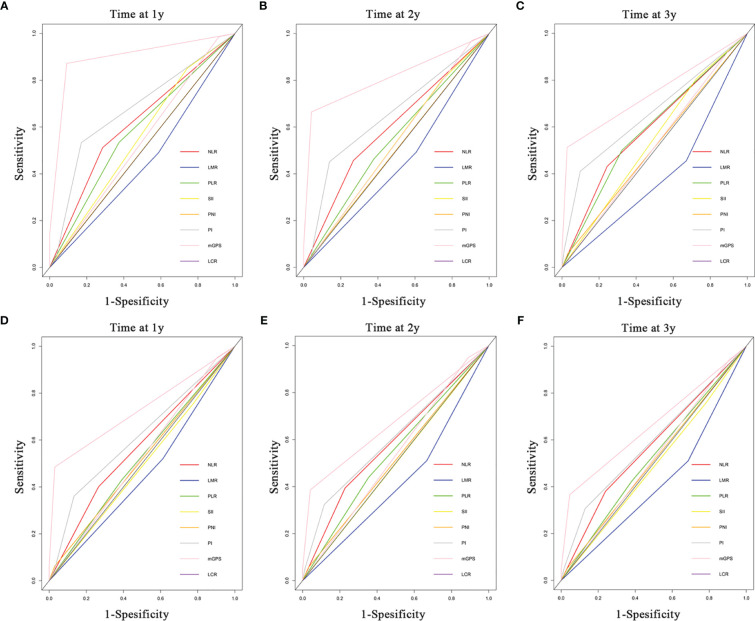
Comparisons of the ROC curves for OS and PFS in the SYSUCC cohort among the inflammation-based score systems. ROC curves of OS at 1 **(A)**, 2 **(B)**, and 3 years **(C)**. ROC curves of PFS at 1 **(D)**, 2 **(E)**, and 3 years **(F)**.

ROC curves and AUROC values were analyzed to contrast the prognostic capacity of eight IBSs in both primary (**Figure 4** and **Table 5**) and validation cohort (**Figure S2** and **Table 5**). The ROC curves were depicted at the 1-, 2-, 3-year follow-ups. C-index was calculated to compare the prognostic power of mGPS to other IBSs. In our primary cohort, the AUROC values of mGPS with OS (1-year 0.897, 2-year 0.813, 3-year 0.743) and PFS (1-year 0.728, 2-year 0.673, 3-year 0.661) were significantly higher than those of any other IBSs (OS: HR, 0.721; 95% CI, 0.705–0.737; all P < 0.001; PFS: HR, 0.645; 95% CI, 0.631–0.659, all P<0.001). The results of the validation cohort presented likewise similarly (OS: HR, 0.651; 95% CI, 0.585–0.717; all P < 0.001; PFS: HR, 0.623; 95% CI, 0.561–0.685; all P<0.005). Thus, the mGPS presented a more powerful prognostic prediction than other IBSs and could divide iCCA patients into subgroups with different survival outcomes more precisely.

**Table 5:** Comparisons of the AUROC values and C-index with mGPS and other IBSs.

Cohort	IBS	OS					DFS				
		AUROC			C-index	P	AUROC			C-index	P
		1 year	2 year	3 year			1 year	2 years	3 years		
SYSUCC cohort	mGPS	0.897	0.813	0.743	0.721 (0.705–0.737)	Ref	0.728	0.673	0.661	0.645 (0.631–0.659)	Ref
	NLR	0.613	0.594	0.594	0.603 (0.579–0.627)	<0.001	0.567	0.583	0.570	0.563 (0.542–0.583)	<0.001
	PI	0.676	0.651	0.657	0.618 (0.599–0.636)	<0.001	0.611	0.605	0.590	0.568 (0.553–0.583)	<0.001
	PLR	0.580	0.543	0.589	0.544 (0.523–0.565)	<0.001	0.519	0.542	0.521	0.529 (0.511–0.547)	<0.001
	SII	0.554	0.528	0.540	0.529 (0.512–0.546)	<0.001	0.490	0.499	0.488	0.504 (0.489–0.519)	<0.001
	PNI	0.501	0.503	0.514	0.503 (0.495–0.511)	<0.001	0.516	0.512	0.509	0.504 (0.497–0.511)	<0.001
	LMR	0.451	0.443	0.392	0.554 (0.533–0.575)	<0.001	0.454	0.422	0.412	0.530 (0.512–0.548)	<0.001
	LCR	0.537	0.532	0.514	0.518 (0.509–0.527)	<0.001	0.524	0.529	0.517	0.516 (0.506–0.527)	<0.001
FHDMU cohort	mGPS	0.683	0.693	0.772	0.651 (0.585–0.717)	Ref	0.613	0.648	0.731	0.623 (0.561–0.685)	Ref
	NLR	0.584	0.545	0.593	0.532 (0.468–0.596)	<0.001	0.563	0.522	0.633	0.534 (0.457–0.593)	<0.001
	PI	0.615	0.619	0.703	0.594 (0.526–0.662)	<0.001	0.552	0.570	0.615	0.562 (0.499–0.625)	0.032
	PLR	0.566	0.527	0.460	0.520 (0.469–0.571)	<0.001	0.506	0.471	0.453	0.498 (0.450–0.546)	<0.001
	SII	0.633	0.598	0.585	0.575 (0.522–0.628)	<0.001	0.578	0.550	0.620	0.562 (0.511–0.613)	0.004
	PNI	0.547	0.510	0.453	0.549 (0.485–0.613)	<0.001	0.499	0.466	0.475	0.525 (0.464–0.586)	0.002

AUROC, area under the ROC curves. Other abbreviations as in **Table 3**.

## Discussion

Over the past decades, various progresses have been made in prophylaxis and treatment of cholangiocarcinoma (1, 2). Nevertheless, the OS and PFS of iCCA patients remained poor (3). For the prediction of prognosis, the TNM grade system has been applied as the mainstream prognostic assessment system since it was presented. However, the TNM grades can only be calculated according to the postoperative pathological factors, and the systemic inflammation level was not included in the TNM grade system. As a result, the TNM grade system cannot make a preoperative overall assessment to guide the therapeutic strategy. To fill this gap, there has been an urgent demand to explore and validate a pre-operative potential prognostic factor for patients with iCCA. IBS, as a combination inflammation index, can objectively reflect the level of inflammation, and further indicate the prognostic and outcomes of cancer patients.

The present study compared the prognostic efficacy of eight common IBSs in patients with iCCA. The univariate and multivariate analyses were further performed to verify the prognostic factors. The mGPS was identified as a significant prognostic factor for predicting OS and PFS in both SYSUCC and FHDMU cohort. Moreover, it was shown that mGPS was superior to the other IBS indexes for predicting the OS and PFS of iCCA patients. It is worth noting that classical pathological elements and TNM staging system made no significant association with the OS and PFS in this study. The probable reasons for the outcome are as follows. First, the powerful predictive performance of mGPS for OS and PFS might mask the role of pathological elements in the multivariate analysis. As a result, the pathological factors showed no significant difference in multivariate analysis. However, that was not intended to deny the predictive effect of pathological elements. As our univariate analysis presented, the TNM staging system was still a statistically significant predictor of prognosis (P = 0.003 in OS, P<0.001 in PFS). Second, the TNM system is a continuously updating and evolving standard with its graded prognostic effect remains controversial. For instance, invasive liver capsule may not adequately reflect the pathogenesis of iCCA tumor, due to the influence of tumor location and tumor size (20). Additionally, the definition of category T3 could barely indicate the biological extent of iCCA tumor (21). Moreover, the number of lymph nodes determined by preoperative imaging examinations and intraoperative findings cannot objectively indicate lymph node metastasis, which may further lead to misjudgment or underestimation of N stage (20, 21). In addition, a further analysis was conducted to elucidate the relationship between mGPS and some clinical and pathological characteristics (**Supplementary Table 1**). The results demonstrated that there was a significant correlation between mGPS and tumor marker (CA19-9, CEA), satellite sites, microvascular invasion, tumor size, lymph node metastasis and TNM stages. These characteristics were significantly related to the poor survival outcomes. Different from these factors, mGPS could be assessed preoperatively. It was worth noting that the CRP level was the key point between mGPS 0 group and mGPS 1/2 groups. In the present study, mGPS 0 group presented a better survival outcome than mGPS1/2 group did. This could also certify the vital role which inflammation played in tumor progression.

The GPS staging system was firstly established in inoperable non-small-cell lung cancer (22), with two major evaluative dimensions: serum ALB and CRP. Serum ALB may indicate the general status as well as the amount of lean tissue of cancer patients. Furthermore, hypoalbuminemia is also associated with cachexia. ALB has been shown to be a prognostic marker in gastric cancer (23) and pancreatic cancer (24), and the role of albumin as a marker of inflammation has been underscored by recent research in malignancy (25). In addition, iCCA, as a type of liver cancer, can weaken the synthesis function of the liver, further leading to the hypoalbuminemia. On the other hand, CRP is not only a sensitive indicator of the systemic inflammatory response. Accumulating evidence indicated the role of CRP in the tumor development and metastasis (26, 27). Theoretically, high CRP level may be due to the production of cytokines from tumor cells (26). As an acute-phase protein, CRP together with IL-6, TNF, and other cytokines further initiates or sustains the systemic inflammatory response (27). Then, inflammation promotes the tumor proliferation, angiogenesis, invasion, and metastasis as a feedback loop (4). It has been confirmed that high level of CRP was correlated with unfavorable survival in esophageal carcinoma (28), colorectal carcinoma (29), as well as multiple myeloma (30). Moreover, as a commonly used index, CRP has high sensitivity and cost-effectiveness and is easily obtained in clinical practice.

With the increasing numbers of studies about GPS and survival in patients with cancers, researchers found that hypoalbuminemia regularly occurred with elevated CRP levels (31). Moreover, the survival outcomes of patients with hypoalbuminemia alone were significantly better than patients with elevated CRP levels, indicating that CRP played a more important role in survival prediction. In case of that, GPS was modified into mGPS (32). Since then, the modified GPS has been occupied in colorectal cancer (32), hepatocellular carcinoma, esophageal cancer (33), and ovarian cancer (34) and simultaneously presented robust prognostic prediction. Significantly, this is the very first study which evaluating the prognostic prediction of the common IBSs in iCCA patients. In this large, multicenter cohort study, we compared the survival curves of these eight frequently used IBSs. Surprisingly, mGPS was not only the independent prognostic factor of OS (P < 0.001) and PFS (P < 0.001) in both of our cohorts, it presented the most powerful performance of prognostic prediction in the common IBSs (all P < 0.005). Similarly, mGPS also presented prominent prognostic manifestations in perihilar cholangiocarcinoma (35) and biliary tract cancer (36) in previous studies. Furthermore, by contrast with the pathological prognostic factors, mGPS, as an inflammation-based score, could make the pre-operative prediction of cancer patients to facilitate accurate stratification and further improve the survival outcomes. Besides, assessments of serum albumin and CRP are simple and inexpensive compared to genetic assessments, which are complicated and expensive.

According to the results of the present study, the treatment strategies of patients with higher mGPS should be optimized. Clinical staff should be especially caution about the indications and contraindications of operation and take careful consideration about the overall healthy situation of these patients. The shorter follow-up intervals were conducive to earlier detection of tumor recurrence or progression. And this would further provide an opportunity for early medical intervention in recurrence. Moreover, the inclusion of routine postoperative chemotherapy in the overall treatment strategies may be beneficial.

Certain limitations of the present study merit discussion. First, the retrospective nature is a potential limitation; we enrolled two cohorts from different regions to restrain this limitation. Second, improvements in perioperative management and treatment methods may lead to the heterogeneous antitumor treatments of our patients’ cohorts and further interfere with the result of the present study. Third, the underlying mechanism of mGPS and poor survival outcome has not been fully demonstrated. Finally, further extensive trans-regional studies were needed to verify the prognostic power of mGPS in iCCA patients.

In conclusion, the present study, we identified mGPS as a sensitive, efficient, simple, rapid, and widely applicable preoperative prognostic factor for iCCA patients. Elevated mGPS scores indicated poor prognosis for these patients. Thus, more effective therapy and frequent surveillance after treatment should be conducted for the iCCA patients with higher mGPS scores.

## Data Availability Statement

The raw data supporting the conclusions of this article will be made available by the authors, without undue reservation.

## Ethics Statement

The studies involving human participants were reviewed and approved by the institutional review board of Sun Yat-sen University Cancer Center and the first affiliated hospital of Dalian Medical University. The patients/participants provided their written informed consent to participate in this study.

## Author Contributions

Study concept: XL. Study design: CH, CZ, and JL. Drafting of the manuscript: CH, CZ, and JL. Data collecting: CH, CZ, JL, CC, and XH. Data analysis: CH, CZ, JL, and XH. Critical revision of the manuscript: XL. All authors contributed to the article and approved the submitted version.

## Funding

This study was supported by the funding of Guangdong Basic and Applied Basic Research Foundation (2020A1515110954) and the funding of Sun Yat-sen University Grant for Medical Humanities Practice and Teaching (no. 23000-18008023).

## Conflict of Interest

The authors declare that the research was conducted in the absence of any commercial or financial relationships that could be construed as a potential conflict of interest.

## Supplementary Material

Supplementary Figure 1 Kaplan-Meier curves for OS **(A)** and PFS **(B)** in patients with iCCA in the FHDMU cohort stratified by the mGPS.

Supplementary Figure 2 Comparisons of the ROC curves for OS and PFS in the FHDMU cohort among the inflammation-based score systems. ROC curves of OS at 1 **(A)**, 2 **(B)**, and 3 years **(C)**. ROC curves of PFS at 1 **(D)**, 2 **(E)**, and 3 years **(F)**.
