# Supplementary figures and images for: Evaluation of Preoperative Inflammation-Based Prognostic Scores in Patients With Intrahepatic Cholangiocarcinoma: A Multicenter Cohort Study
Source: Front Oncol. 2021 Jun 17;11:672607. doi: 10.3389/fonc.2021.672607 (PMC8247471; doi:10.3389/fonc.2021.672607)

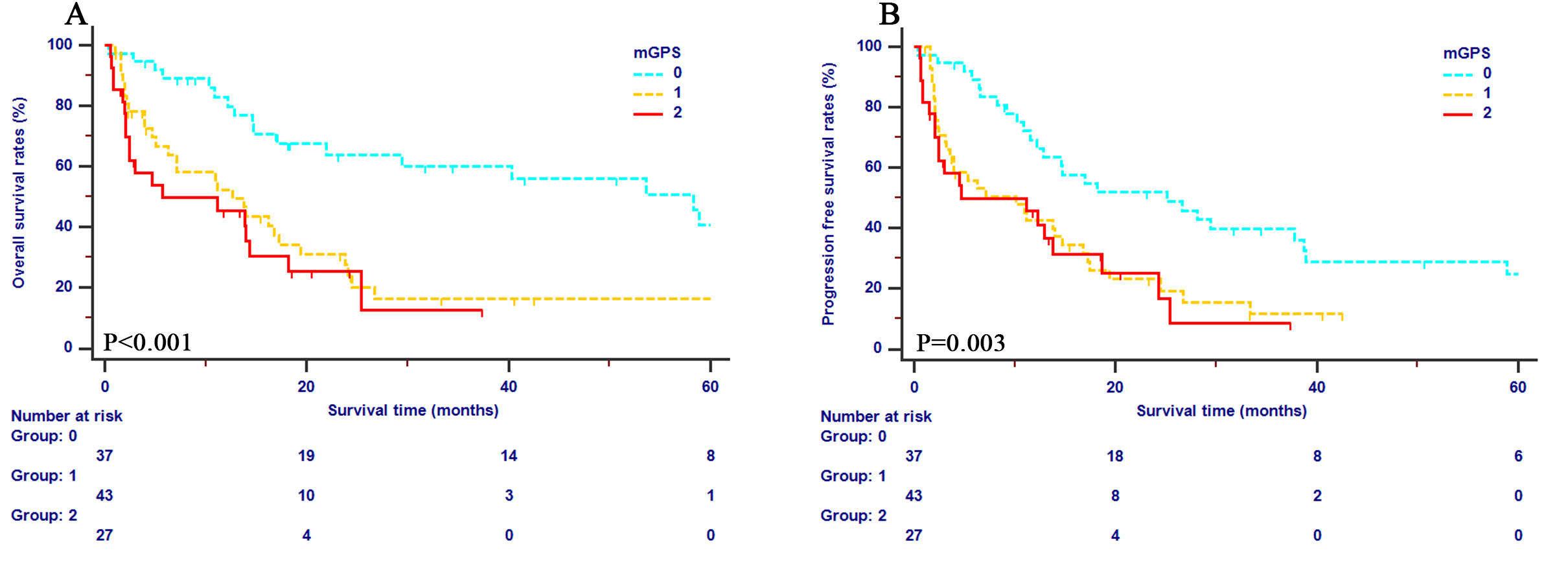

Supplement: Supplementary Figure 1 — Kaplan-Meier curves for OS (A) and PFS (B) in patients with iCCA in the FHDMU cohort stratified by the mGPS. [file Image_1.tif]

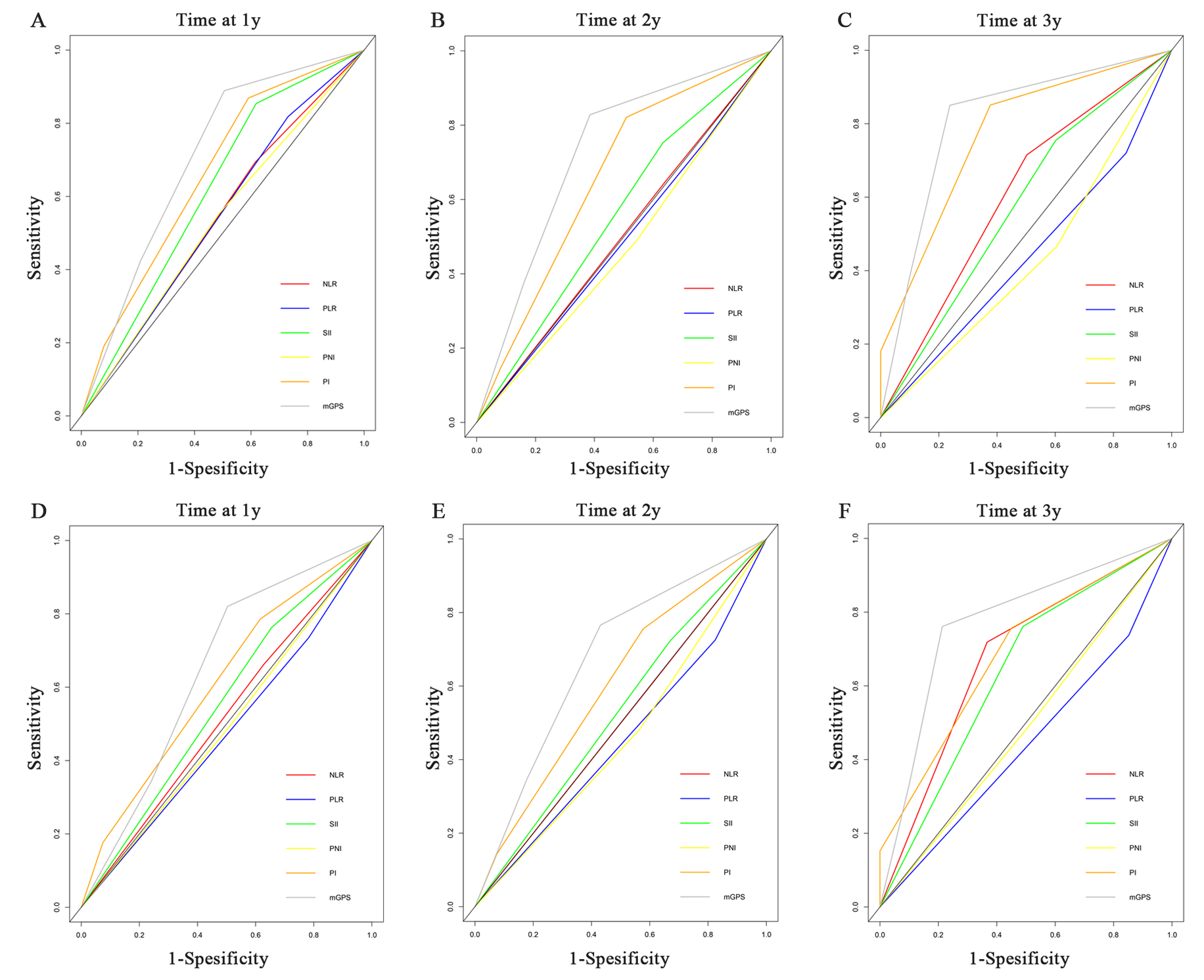

Supplement: Supplementary Figure 2 — Comparisons of the ROC curves for OS and PFS in the FHDMU cohort among the inflammation-based score systems. ROC curves of OS at 1 (A), 2 (B), and 3 years (C). ROC curves of PFS at 1 (D), 2 (E), and 3 years (F). [file Image_2.tif]
