# Supplementary material for: Evaluation of Preoperative Inflammation-Based Prognostic Scores in Patients With Intrahepatic Cholangiocarcinoma: A Multicenter Cohort Study
Source: Front Oncol. 2021 Jun 17;11:672607. doi: 10.3389/fonc.2021.672607 (PMC8247471; doi:10.3389/fonc.2021.672607)
Supplement: Supplementary file 3 [file Table_1.docx]

| Supplementary Table 1. Correlation between mGPS subgroups and significant clinical characteristics. | | | | |
| --- | --- | --- | --- | --- |
| characteristic | mGPS 0 | mGPS 1 | mGPS 2 | p |
| CA19-9 (U/ml) |  |  |  | 0.002 |
| ≤ 35 | 115 | 26 | 0 |  |
| >35 | 101 | 41 | 9 |  |
| CEA (ng/ml) |  |  |  | <0.001 |
| ≤ 5 | 172 | 36 | 3 |  |
| > 5 | 44 | 31 | 6 |  |
| Satellite sites |  |  |  | 0.002 |
| Absence | 161 | 35 | 5 |  |
| Presence | 55 | 32 | 4 |  |
| Thrombus |  |  |  | 0.327 |
| Absence | 202 | 59 | 8 |  |
| Presence | 14 | 8 | 1 |  |
| Tumor differentiation |  |  |  | 0.156 |
| Low | 5 | 1 | 0 |  |
| Medium | 83 | 22 | 0 |  |
| High | 128 | 44 | 9 |  |
| Microvascular invasion |  |  |  | <0.001 |
| Absence | 182 | 53 | 2 |  |
| Presence | 34 | 14 | 7 |  |
| Lymph-vessel invasion |  |  |  | 0.265 |
| Absence | 204 | 60 | 9 |  |
| Presence | 12 | 7 | 0 |  |
| Macrovascular invasion |  |  |  | 0.098 |
| Absence | 205 | 62 | 7 |  |
| Presence | 11 | 5 | 2 |  |
| Tumor size |  |  |  | <0.001 |
| ≤ 5cm | 104 | 11 | 0 |  |
| ≤ 5cm | 112 | 56 | 9 |  |
| LN metastasis |  |  |  | <0.001 |
| Absence | 197 | 48 | 5 |  |
| Presence | 19 | 19 | 4 |  |
| TNM 8^th^ |  |  |  | 0.002 |
| I | 29 | 5 | 0 |  |
| II | 56 | 17 | 0 |  |
| III | 131 | 45 | 9 |  |
